# Approaching hemophagocytic lymphohistiocytosis

**DOI:** 10.3389/fimmu.2023.1210041

**Published:** 2023-06-22

**Authors:** Aurora Chinnici, Linda Beneforti, Francesco Pegoraro, Irene Trambusti, Annalisa Tondo, Claudio Favre, Maria Luisa Coniglio, Elena Sieni

**Affiliations:** ^1^ Department of Neurosciences, Psychology, Drug Research and Child Health (NEUROFARBA), University of Florence, Florence, Italy; ^2^ Department of Pediatric Hematology Oncology, Meyer Children’s Hospital IRCCS, Florence, Italy; ^3^ Department of Health Sciences, University of Florence, Florence, Italy

**Keywords:** HLH - hemophagocytic lymphohistiocytosis, FHL – familial hemophagocytic lymphohistiocytosis, perforin, degranulation, flow cytometric assay, NGS - next generation sequencing

## Abstract

Hemophagocytic Lymphohistiocytosis (HLH) is a rare clinical condition characterized by sustained but ineffective immune system activation, leading to severe and systemic hyperinflammation. It may occur as a genetic or sporadic condition, often triggered by an infection. The multifaceted pathogenesis results in a wide range of non-specific signs and symptoms, hampering early recognition. Despite a great improvement in terms of survival in the last decades, a considerable proportion of patients with HLH still die from progressive disease. Thus, prompt diagnosis and treatment are crucial for survival. Faced with the complexity and the heterogeneity of syndrome, expert consultation is recommended to correctly interpret clinical, functional and genetic findings and address therapeutic decisions. Cytofluorimetric and genetic analysis should be performed in reference laboratories. Genetic analysis is mandatory to confirm familial hemophagocytic lymphohistiocytosis (FHL) and Next Generation Sequencing is increasingly adopted to extend the spectrum of genetic predisposition to HLH, though its results should be critically discussed with specialists. In this review, we critically revise the reported laboratory tools for the diagnosis of HLH, in order to outline a comprehensive and widely available workup that allows to reduce the time between the clinical suspicion of HLH and its final diagnosis.

## 1 Introduction

Hemophagocytic Lymphohistiocytosis (HLH) is a rare clinical condition characterized by sustained but ineffective immune system activation, leading to severe and systemic hyperinflammation. It may develop in the context of a familial disorder (FHL) or as a sporadic condition, in association with diverse triggers, such as infections, autoinflammation, acquired immune deficiencies, or malignancies. The identification of familial cases is crucial to allow a definitive cure by hematopoietic stem cell transplantation (HSCT). Clinical manifestations may include persistent fever, hepatosplenomegaly, multi lineage cytopenia, coagulopathy, hyperferritinemia, and central nervous system involvement. These signs and symptoms are the consequence of an exaggerated cytokine release and organ infiltration by activated lymphocytes and macrophages. Patients may rapidly deteriorate and develop multi organ failure and death. Thus, prompt recognition and treatment are essential for patients’ survival.

Historically, FHL has been associated to impaired cytotoxicity due to biallelic mutations in FHL-related genes. Nowadays, the advent of Next Generation Sequencing (NGS) and improvement of functional studies led to re-define HLH as a syndrome with etiopathogenetic heterogeneity, blowing the distinction between familial and sporadic cases. Starting from an historical perspective up to the current understanding of HLH, in this review we critically revise this complex condition and the available diagnostic tools, with the final aim to describe a comprehensive clinical and laboratory approach for the management of patients with suspected HLH.

## 2 Unveiling HLH

**Figure 1:**
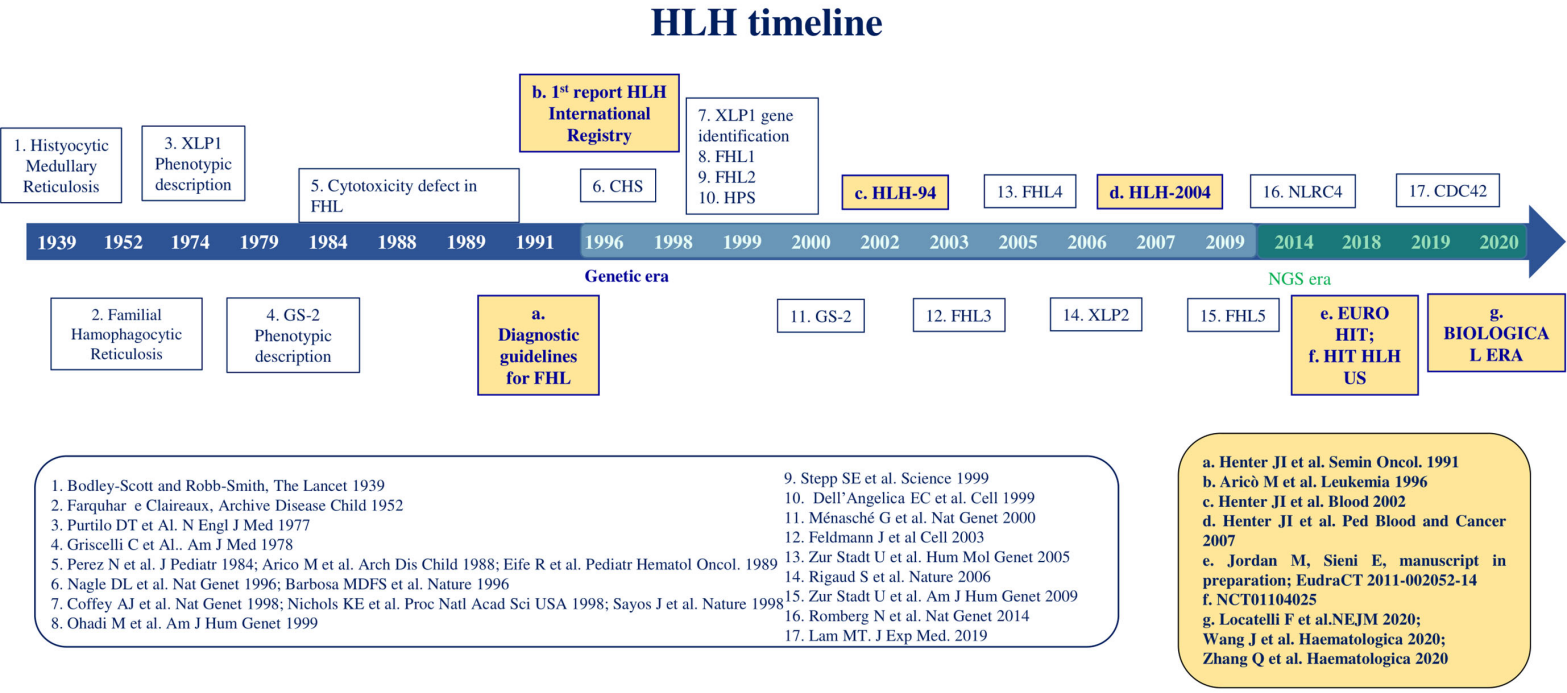
HLH timeline. The figure displays the main steps in HLH discovery along the last 80 years.

The first description of HLH dates 1939, when Scott and Robb-Smith reported 4 patients with unremitting fever, lymphadenopathy, and hepatosplenomegaly (1) (**Figure 1**). A few years later, Farquhar and Claireaux reported two siblings with a similar clinical syndrome. This was the first description of the familial form of the disease (2). In the next decades, similar features were observed both in familial clusters and as sporadic forms. In 1979, Risdall et al. described 19 patients with evidence of HLH and viral infection after transplantation, later defined ‘Virus associated hemophagocytic syndrome’ to denote any case of HLH without a genetic cause. Since other infective agents were shown to trigger HLH, these conditions were redefined as ‘infection associated hemophagocytic syndrome’ (IAHS) (3). The term ‘Macrophage activation syndrome’ (MAS) was firstly used in 1992 by Albert et al. to refer to a condition characterized by cytopenias, organ dysfunction, coagulopathy, and inappropriate activation of macrophages in a proinflammatory milieu (4).

The initial identification of a biological marker refers to the impairment of natural killer (NK) cell cytotoxic activity, which was observed in affected patients, compared with healthy relatives, in the 1980s (5–8). The first gene related to FHL, *PRF1*, was identified in December 1999 and *PRF1*-mutated FHL was then named FHL type 2 (FHL-2) (9). FHL-1 had been previously associated with an unknown gene mapping in position 9q21.3-2 (10). At the same time, mutations in *SH2D1A* affecting the SLAM associated protein (SAP) signaling have been recognized as the cause of a clinical syndrome phenotypically described in 1977 by Purtilo et al. (X-linked lymphoproliferative syndrome type 1, XLP-1) (2). Since then, the study of familial cases allowed the recognition of genetic alterations directly affecting proteins involved in cytotoxic activity, such as Munc13-4 (FHL-3), Syntaxin 11 (FHL-4), Munc18-2 (FHL-5), RAB27A (Griscelli Syndrome type 2, GS2), LYST (Chediak-Higashi Syndrome, CHS) (11–15). Lately, other variants were linked to dysregulated or persistent inflammasome activation involving XIAP, NLRC4 and CDC42 proteins (16). In recent years, NGS analysis led to the identification of an increasing number of genes predisposing to HLH.

Diagnostic guidelines were defined for the first time in 1991 and included fever and splenomegaly, hemophagocytosis in bone marrow, spleen, or lymph nodes, cytopenias, hypertriglyceridemia and/or hypofibrinogenemia (17). At that time, the first report of the international Registry of HLH on 122 patients reported a 5-year survival of only 21% (18). In January 1995, the HLH Study Group of the Histiocyte Society opened its first international treatment study, HLH-94, consisting of corticosteroids and the antineoplastic etoposide, followed by hematopoietic stem cell transplantation (HSCT) for patients with FHL (19). This trial led to a dramatic improvement of outcome up to a 5-year overall survival of 54%. Some years later, the HLH-2004 protocol showed that intensification with cyclosporine A upfront and intrathecal corticosteroids did not significantly improve the outcome, resulting in an OS of 62% (19, 20). With the HLH-2004 protocol the diagnostic criteria were updated with the inclusion of low or absent NK-cell activity, hyperferritinemia and high levels of soluble IL-2 receptor α chain (sIL-2r, also named sCD25) (21). In 2007, an alternative immunotherapeutic approach with antithymocyte globulin (ATG) and methylprednisolone followed by consolidation and HSCT was proposed, based on a single-center experience. It resulted in a higher and faster response rate at 8 weeks compared to HLH-94 at the price of a higher reactivation rate, ultimately not affecting OS (22). A combination between the HLH-94 and the ATG protocol (EURO-HIT-HLH, EudraCT 2011-002052-14) was then investigated, not resulting in significant improvement of outcomes (Jordan M., Sieni E., in preparation). Alternative or salvage treatment approaches have been proposed and are currently under investigation. Among them, monoclonal antibodies anti-CD52 (alemtuzumab), anti-interferon-γ (emapalumab) and anti-Janus Kinases (ruxolitinib) showed preliminary efficacy and are currently under investigation (23–25). Finally, preclinical studies on T cell-mediated gene therapy, by both lentiviral or gamma retroviral vectors, are promising for a definitive cure of FHL-2, FHL-3 and XLP-1 (26–28).

## 3 Cataloging HLH: a tool or a trap?

Traditionally, HLH is classified into primary and secondary. Primary HLH, or FHL, is caused by biallelic mutations in genes involved in the secretory lysosome-dependent exocytosis pathway and usually manifests in early infancy. The following FHL subtypes have been described: FHL-2 due to mutations in *PRF1*, FHL-3 (*UNC13D)*, FHL-4 (*STX11)*, FHL-5 (*STXBP2)*, Griscelli Syndrome type 2 (*RAB27A)* and Chediack-Higashi syndrome (*LYST)* (9–15). Secondary HLH, also referred to as sporadic, which is more frequent in older children and adults, may develop in the context of infections, malignancies, rheumatic diseases, chemotherapy, or transplantation (29). In the setting of a rheumatologic condition, secondary HLH is referred to as MAS, a potentially life-threatening complication of systemic inflammatory disorders dominated by an overwhelming inflammation. It occurs mostly in in systemic juvenile idiopathic arthritis (JIA), but it is also described in other autoimmune or autoinflammatory conditions, including Systemic Lupus Erythematosus (SLE), Adult onset Still disease (AOSD), Kawasaki disease and periodic fever syndromes (30).

More recently, an increasing number of adults have been diagnosed with primary HLH (31) and monoallelic mutations of FHL-related genes have been identified in patients with HLH and MAS (32, 33). This blurred the distinction between primary and secondary HLH and prompted the hypothesis of HLH as a continuum where different weights of genetic and environmental factors contribute to the development of HLH. Indeed, biallelic disruptive mutations in FHL-related genes lead to severe cytotoxic defects and early onset FHL, even in the absence of environmental triggers, while hypomorphic or monoallelic mutations in the same genes are associated to a less complete cytotoxic defect and a later onset of the disease, as they require a more powerful trigger to elicit the HLH phenotype (34). In the last decade, the advent of NGS, from target resequencing to whole exome sequencing (WES), exponentially enhanced the ability to identify a growing number of variants, by expanding the spectrum of genetic predisposition to HLH (**Table 1**). Biallelic *RHOG* loss-of-function variants were recently reported in a patient with early-onset HLH and defective lymphocyte exocytosis but reports of additional patients are needed to gain insights into penetrance (35). Besides variants in genes required for lymphocyte cytotoxicity, mutations leading to inflammasome activation (i.e. autosomal dominant mutations in *NLRC4* or hemizygous mutations in *XIAP*) and mutations involved in immune response to viral infections (i.e. hemizygous mutations in *SAP*) have been related to HLH (36, 37). Recently, heterozygous mutations in *CD48* have also been associated to HLH (38) as well as biallelic *ZNFX1* loss-of-function variants that have been reported in three families with HLH or HLH-like disease (39).

**Table 1:** Genetic alterations with HLH as a predominant manifestation.

Pathogenic pathway	Gene	Protein	OMIM	Disorder	Special clinical features	MOI
**Cytotoxicity defect**	*PRF1*	Perforin	603553	FHL-2		AR
	*UNC13D*	Munc 13-4	608898	FHL-3		AR
	*STX11*	Syntaxin 11	603552	FHL-4		AR
	*STXBP2*	Munc 18-2	613101	FHL-5	Gastrointestinal symptoms, hypogammaglobulinemia	AR
	*RAB27A*	Rab27a	607624	Griscelli Syndrome Type 2 (GS-2)	Partial albinism, CNS symptoms	AR
	*LYST*	Lyst	214500	Chediak Higashi Syndrome (CHS)	Partial albinism, neutropenia	AR
	*AP3B1*	AP3B1	608233	Hermansky-Pudlak Syndrome Type 2 (HPS2)	Partial albinism, bleeding	AR
**Impaired control of infections**	*SH2D1A*	SAP	308240	X-linked Lymphoproliferative Syndrome Type 1 (XLP-1)	HLH, EBV 40%, lymphoproliferation, hypogammaglobulinemia, aplastic anemia, vasculitis	XLR
	*ITK*	ITK	613011	Lymphoproliferative syndrome 1		AR
	*MAGT1*	MAGT1	300853	Immunodeficiency, XL, magnesium defect, EBV infection and neoplasia		XL
	*CD27*	CD27	615122	Lymphoproliferative syndrome 2		AR
	*CD70*	CD70	618261	Lymphoproliferative syndrome 3		AR
	*CTPS1*	CTPS1	615897	Immunodeficiency 24		AR
	*RASGRP1*	RAS guanyl-releasing protein 1	618534	Immunodeficiency 64		AR
	*ZNFX1*	ZNFX1	619644	Immunodeficiency 91 and hyperinflammation		AR
**Dysregulated inflammasome activation**	*XIAP*	XIAP	300635	X-linked Lymphoproliferative Syndrome Type 2 (XLP-2)	HLH, EBV 30%, IBD, splenomegaly hypogammaglobulinemia, recurrent infections, uveitis, periodic fever	XLR
	*NLRC4*	NLRC4	616050		Autoinflammation with infantile enterocolitis	AD
	*CDC42*	CDC42	116952		Neonatal onset of pancytopenia, autoinflammation, rash, HLH (NOCARH)	AD

AD, Autosomal dominant; AD, Autosomal recessive; XLR, X-Linked Recessive.

On these bases, HLH can be now considered as a dynamic continuum of inflammatory conditions, where the risk of developing the syndrome is the result of the subtle balance between a predisposed genotype and environmental factors (33, 40).

Trying to divide patients with HLH into primary and secondary forms is likely to trap clinicians in a conceptual labyrinth that could lead to unrecognition or misclassification of blurred cases. Integrating the concept of HLH as a continuum with a deep genetic understanding will improve the management of patients with HLH.

## 4 HLH diagnosis: how to compose the puzzle

HLH diagnosis is challenging due to clinical features overlapping with other conditions, including sepsis and severe inflammation (41). Although regularly adopted in clinical settings, the HLH-2004 criteria were developed in the context of a pediatric clinical trial. For this reason, despite their undeniable utility, they show some limitations. Firstly, they include non-specific markers of a common inflammatory phenotype, with particular emphasis on features that are likely related to HLH but are also found in other conditions. For example, the cutoff defined for ferritin levels (>500 ng/ml) may indicate a multiplicity of diseases characterized by inflammation, whether a threshold over 2000 ng/mL resulted in 70% sensitivity and 68% specificity in a cohort of 123 patients with HLH (42). Another pitfall is the inclusion of NK cell cytotoxicity assay, which was demonstrated to have poor sensitivity and specificity (60% and 72%, respectively), and it is now overcome by flow cytometry screenings (43). Finally, bone marrow aspirate before treatment initiation is mandatory to rule out hematological malignancies, whereas the absence of hemophagocytosis should not exclude HLH since it is detected in only 40% of patients at disease onset (HLH Italian Registry data 2014-2022, unpublished data). Despite these limitations, occurring when criteria are taken individually, their combinatory evaluation is still useful for a first clinical suspect and to monitor disease activity. In the attempt to simplify disease recognition, some years ago we proposed the combination of fever, splenomegaly, thrombocytopenia and hyperferritinemia as a minimal set of clinical criteria able to address the clinician to HLH suspicion, by observing a specificity of 84% in a cohort of FHL3 patients (44). Though not specific, high ferritin levels strongly support the HLH suspicion, while normal ferritin values point to other diagnosis.

HLH-2004 criteria are mainly useful to identify paediatric FHL, but show poor sensitivity for secondary HLH, MAS or other inflammatory conditions (45). To fill this gap, 2016 classification criteria for MAS complicating sJIA were validated (30). These criteria were developed by expert consensus and real-life data, by premising that clinical symptoms of suspected MAS are scarcely specific and may be deferred over time. On these bases, the only clinical symptom included was fever, leaving room to laboratory tests (ferritin, platelet count, AST, fibrinogen, and triglycerides). The validation of 2016 classification criteria resulted in a discrimination of MAS from other conditions with a sensitivity of 0.73, a specificity of 0.99, a positive predictive value of 97.4% and a negative predictive value of 85.9% (30). These criteria show two major limitations: on the one hand, they are reliable only on patients with sJIA/MAS, on the other, they were developed as classification criteria and cannot be considered diagnostic. To further simplify the distinction of MAS in the setting of sJIA from sJIA flares, a cut point interval for the ratio of ferritin to the erythrocyte sedimentation rate (ESR) was also proposed (46). Additionally, HScore was developed for adult cohorts on the basis of weighted clinical, biochemical, and cytological variables that were comparable to HLH-2004 criteria. The authors indicated a median score of 230 (IQR 203-257) in HLH positive cases and 125 (IQR 91–150) in negative cases (47). This scoring showed a 90% sensitivity and 79% specificity in adults (48). It is suitable to identify secondary HLH in general, but it is not validated in a pediatric cohort and was developed in the setting of patients with primarily malignancy related HLH.

In summary, the scores reported above and charted in **Table 2** are a powerful tool for clinicians to rapidly identify patients with HLH allowing a prompt treatment start.

**Table 2:** Comparison between HLH-2004 criteria, HScore and MAS classification criteria.

Criteria	HLH-2004	2016 MAS Classification	HScore
*Fever (°C)*	≥ 38.5	Present	>39.4: 49 points 38.4-39.4: 33 points
*Organomegaly* *Splenomegaly: S* *Hepatomegaly:H*	S		H and S: 38 points H or S: 23 points
*Cytopenia* *Hemoglobil: Hb (g/dL)* *Platelet: Plt (x 10^9/^L)* *Neutrophil: Nt (x 10^9/^L)*	C ≥ 2 series Hb< 9 Plt< 100 Abs Nt< 1	Plt ≤ 181	3 series: 34 points 2 series: 24 points
*Triglycerides (mmol/L)*	≥ 3.0	>1.76	> 4.0: 64 points 1.5 – 4.0: 44 points
*Fibrinogen (mg/dL)*	≤ 150	≤ 156	≤ 2.5: 30 points
*Ferritin (µg/L)*	≥ 500	>684	> 6: 50 points 2-6: 35 points
*Serum AST (IU/L)*		>48	≥ 30: 19 points
*Hemophagocytosis*	Hemophagocytosis in bone marrow, spleen or lymph nodes		Present: 35 points
*Immunosuppression*			Present: 18 points
*NK cell activity*	Low or absent		
*sCD25 (sIL2Rα, U/ml)*	≥ 2400		
*Age at onset (years)*			

## 5 Specialized diagnostic tests

**Figure 2:**
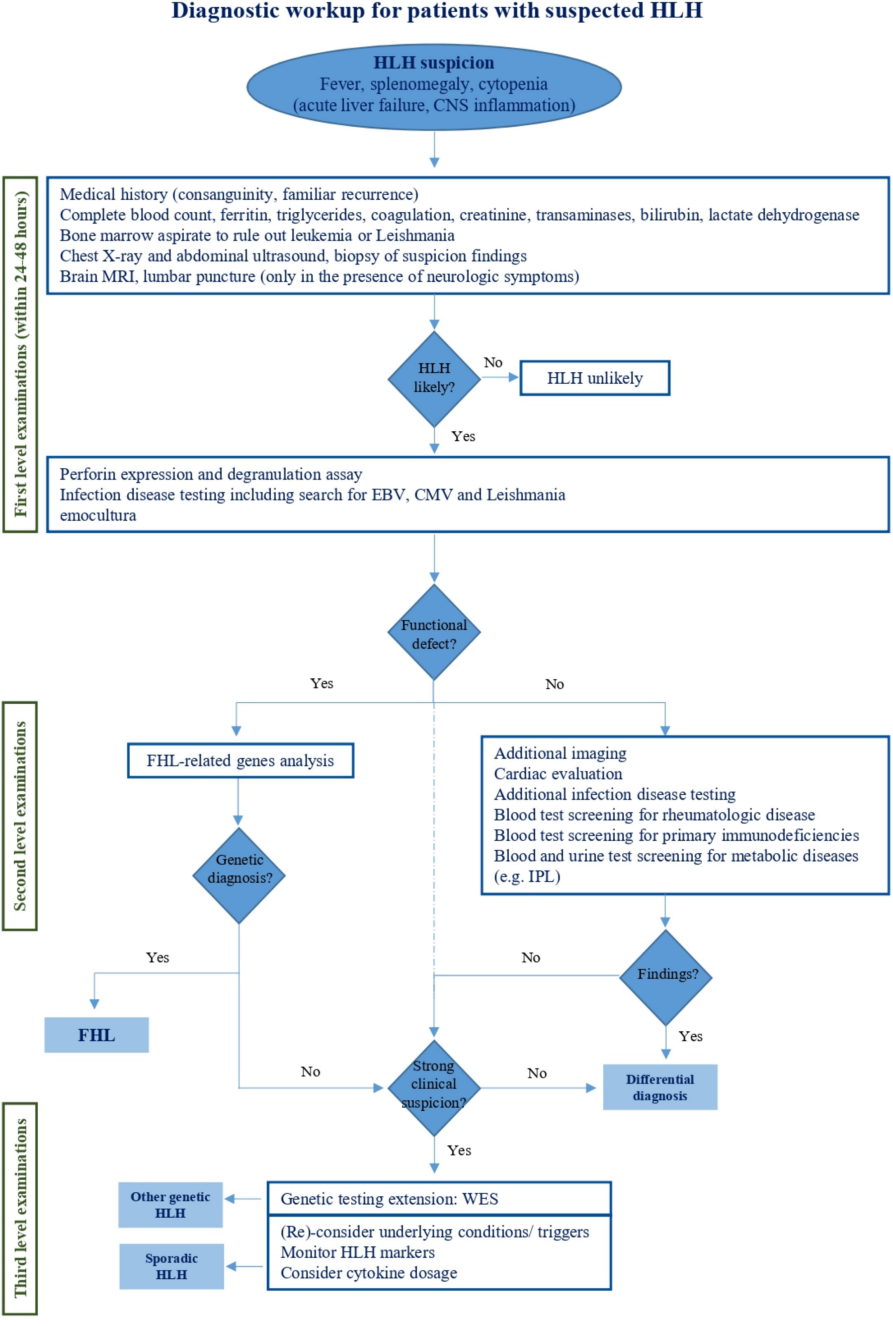
Diagnostic workup for patients with suspected HLH. This figure reports the management of HLH handling from initial clinical suspicion to specialized diagnostic tests aimed at HLH differential diagnosis.

Once HLH is suspected, first line examinations are requested to confirm it (**Figure 2**). Specialized laboratory tests are indeed supportive for the diagnosis of the syndrome and the monitoring of disease activity, but their integration with clinical data is decisive. Here, we report the available laboratory tests, some of which are increasingly used in clinical practice, while others are available for research only (**Table 3**).

**Table 3:** Main diagnostic markers in HLH.

Diagnostic marker	Clinical significance
Hyperferritinemia	Marker of Hyperinflammation/HLH/MAS
Increased sCD25 (sIL-2r)	T cell-driven HLH/MAS
Increased CXCL9	IFN-g-driven HLH/MAS
Increased IL-18	MAS, NLRC4, XLP-2
Increased Ferritin/ESR ratio	MAS vs. sJIA without MAS
Increased IL-18/CXCL9	MAS vs. FHL
Increased CD38/HLA-DR expression	T cell-driven HLH
Increased HLA-DR expression	T cell-driven HLH
Increased sCD163	Macrophage activation/MAS
Impaired Perforin expression	FHL-2
Impaired Degranulation assay	FHL 3-5, GS-2, CHS, HPS-2
Impaired NK cell cytotoxicity	FHL 2-5, GS-2, CHS, HPS-2
Defective SAP expression	XLP-1
Reduced 2B4 receptor inhibitory effect	XLP-1
Defective XIAP expression	XLP-2
Impaired NOD2 signaling	XLP-2

### 5.1 Perforin detection and degranulation assay

In physiologic conditions, activation and priming of NK and cytotoxic T cells (CTLs) by a target cell stimulate the formation of secretory lysosomes, which are delivered to the intercellular space by exocytosis. These organelles contain cytotoxic proteins such as perforin and granzymes (16). Perforin is a membranolytic protein stored in specialized cytoplasmic granules of NKs and CTLs. Its ability to form pores on target cell membranes is crucial for the resultant delivery of granzyme B, which in turn is responsible for target cell apoptosis by osmotic lysis (49). Biallelic mutations in *PRF1* result in reduced or absent cytolytic effect on target cells by CTLs and NKs with the consequent exacerbation of pro-inflammatory conditions (50).

A rapid and reliable method to detect perforin defects is protein intracellular staining and signal detection by flow cytometry. Kogawa et al. validated a protocol by examining a cohort of pediatric and adult patients with the aim to correlate *PRF1* mutations with reduced protein levels (50). In healthy controls, the percentage of NK cells expressing perforin was 86% ± 5% in pediatric patients (1-15 years) and 92% ± 6% in subjects older than 15 years. In contrast, FHL-2 patients showed partial or complete deficiency of protein expression (MCF cut-off <38). The analysis was extended to parents and asymptomatic siblings and, with rare exceptions, there was a coincidence between defective perforin expression and genetic alterations (43, 50, 51). Thus, if well standardized, the flow-cytometric intracellular detection of perforin is rapid and accurate for identifying FHL-2 patients, with a sensitivity and specificity of 96.6% and 99.5%, respectively (43).

Degranulation assay enables to investigate the capacity of NK cells to pour the content of cytotoxic granules in the extracellular space. Unstimulated NK cells express the CD107a protein anchored to the membrane of intracytoplasmic granules. Upon activation, granules fuse with the outer cell membrane by exposing CD107a on the cell surface (52). Biallelic mutations in genes coding for proteins involved in degranulation machinery (namely *UNC13D*, *STXBP2*, *STX11*, *RAB27A*, *LYST*, and *AP3B1*, but not *PRF1*, *SH2D1A* and *BIRC4*) result in the reduction of CD107a externalization after proper stimulation *in vitro*, by resulting in cytotoxicity defects (43, 51, 53). For this reason, flow cytometric detection of surface CD107a expression represents an interesting instrument to rapidly screen HLH suspected patients. Independent studies reported a sensitivity between 93.8 and 96% and a specificity between 73 and 88% in the detection of patients with biallelic gene mutation in the degranulation pathway (43, 51, 52). Despite the assay being widely used in HLH specialized centers following the protocol by Marcenaro et al. (52), technical details may vary in different laboratories due to local needs (namely different culture medium and/or recombinant cytokines brands, different incubation time). This scenario should be considered in comparing results from different laboratories and highlight the importance of complementary genetic analysis.

### 5.2 NK cell cytotoxicity test

NK cell cytotoxicity assay was included in HLH-2004 diagnostic criteria and has been considered a valid test for the screening of patients carrying biallelic mutations in *PRF1*, *UNC13D*, *STXBP2*, *STX11*, *RAB27A*, *LYST*, and *AP3B1*, but not those with mutations in *SH2D1A* and *XIAP/BIRC4*.

NK-mediated immune surveillance consists of several sequential steps: target-cell identification, effector cell activation, cytolytic granule secretion and target-cell lysis. Killing decision results from a complex integration of intracellular pathways within the NK cell, mainly deriving from activating and inhibitory receptors. Tumor- and virus-infected cells express damage-related molecules that bind to activating receptors; at the same time, they downregulate MHC class I molecules, depriving inhibitory NK receptors of their proper stimuli. The result is the activation of NK-mediated cytotoxicity. The cytotoxicity assay evaluates, by flow-cytometry, the killing of carboxyfluoresceinsuccinimidyl ester (CFSE)-labeled K562 target cells when co-cultured with NK cells at different ratios (54). Unfortunately, the test is labor intensive since it requires radioactivity and makes use of scarcely available sources such as NK cells and ^51^Cr isotope (43). Furthermore, it cannot distinguish between primary and secondary HLH. As reported by Rubin et al, NK cytotoxicity has similar specificity, but less sensitivity compared to CD107a evaluation (59.5% and 72.0% versus 93.8% and 73.0%, respectively) and is less reliable than the perforin assay (96.6% and 99.5%) (43). For these reasons, and thanks to their higher approachability, perforin expression and degranulation assay are now widely preferred to cytotoxicity assays.

### 5.3 SAP and XIAP expression and functional assay

Patients harboring *SH2D1A* mutations usually do not display defects in the degranulation machinery. The *SH2D1A* gene encodes the SLAM-associated protein, also called SAP, which is principally expressed in NK and T cells, and is likely to regulate signal transduction initiated by SLAM protein family (55). Among them, 2B4 is a surface protein which recruits SAP upon recognition of CD48. If SAP is defective or dysfunctional, 2B4 acts as inhibitory protein, by impairing NK and T cells ability to face infections, EBV upon others (56, 57). Since EBV particularly targets B cells, the main feature of SAP defective patients is B cells accumulation, with severe manifestations ranging from persistence of inflammation to fulminant mononucleosis or B-cell lymphomas (58, 59). It has been demonstrated that *SH2D1A* missense mutations may lead to a decrease in SAP protein expression (60). Flow-cytometry evaluation of SAP expression has been proposed as a rapid diagnostic assay to screen XLP-1 patients; a validated and specific rat (KST-3) and, more recently, a murine (1C9)14 anti-SAP mAb have been made available (61). However, these antibodies fail to recognize missense mutation not affecting SAP expression.

Accordingly, the flow cytometric combinatorial evaluation of intracellular SAP expression and 2B4 receptor inhibitory effect was proposed. Indeed, in patients whose clinical picture suggests an XLP-1 syndrome, the sensitivity of SAP detection resulted insufficient, while 2B4 receptor assay was highly predictive by resulting dysfunctional in all XLP-1 patients and normal in healthy controls, as well as non-XLP-1 patients with persistent fever due to EBV infection (62).

A rare form of immunodeficiency often resulting in HLH is the deficiency of X-linked Inhibitor of Apoptosis (XIAP), induced by alterations in the *XIAP/BIRC4* gene (63). Less commonly than XLP-1, XLP-2 may be triggered by EBV infection (29). Furthermore, XIAP deficiency was associated with a particular inflammatory pattern consisting of recurrent fever, uveitis, and inflammatory bowel disease (IBD) (64). To detect XIAP protein levels, Marsh et al. proposed a flow cytometric analysis of intracellular XIAP expression in peripheral blood lymphocytes. Interestingly, the test was accurate and fast in recognizing protein absence in all patients harboring different *XIAP/BIRC4* mutations that resulted in protein deletion (65). However, since some missense mutations may not be unveiled by XIAP protein detection, a more recent study suggested to investigate nucleotide-binding and oligomerization domain 2 (NOD2) signaling, which was shown to be part of the XIAP downstream pathway in the activation of MAP kinases and IκB kinase complex (IKK). The activation of this pathway results in the production of cytokines such as tumor necrosis factor (TNF-α), IL-1β, IL-6 and IL-8 (66, 67). Since impaired NOD2 signaling is peculiar when XIAP is deficient, Ammann et al. developed an intracellular flow cytometry functional assay by stimulating NOD2-mediated TNF-α production with muramyl dipeptides (MDP), a cell component of bacterial peptidoglycans. They analyzed 12 patients carrying different *XIAP/BIRC4* mutations and found a marked reduction of TNF-α production in all of them, when compared with healthy donors and healthy carrier mothers. On the other hand, XIAP deficiency was observed by flow cytometry in 10/12 patients. The proposed functional assay had a higher sensitivity and was more reliable than XIAP expression analysis (68).

### 5.4 HLA-DR and CD38 expression

The role of T cells in HLH was widely explored in the past decades, reporting T-cell hyperactivation as the main feature of primary HLH (69). Indeed, it was observed that MHC class II molecules, such as HLA-DR, are not expressed on naive human T cells, but they can be induced by cytokines through a TCR mediated activation (70). Increased HLA-DR expression on T cells were observed in pediatric cohorts affected by HLH (71). In 2017, Ammann et al. performed flow cytometric analysis of HLA-DR expression in CD4^+^ and CD8^+^ T cells from a cohort of 93 patients with active HLH (72). While HLA-DR expression in CD8^+^ T cells was below 12% in healthy donors, it ranged from 21% in secondary patients with HLH with no underlying infections to 61.5% and 64.4% in patients with virus-triggered secondary HLH and primary HLH, respectively. The calculated sensitivity was above 80%. The inability to distinguish between primary and secondary HLH was overcome by the analysis of the perforin-expressing CD4^+^ T cell subset, which was activated only in primary HLH (72).

CD38 is a multi-lineage surface marker involved in cell activation and proliferation (73). Recently, the subpopulation of CD8^+^ CD38^high^/HLA-DR^+^ cells was investigated as a diagnostic marker for HLH in a cohort of 43 pediatric patients (median age 2.6 years) without rheumatologic or malignancy conditions (74). The presence of CD8^+^/CD38^high^/HLA-DR^+^ T cells was distinctly found in patients with HLH, as compared to patients with sepsis. The percentage of CD38^high^ cells in CD3^+^ CD8^+^ subset was also followed-up in two patients and showed a reduction following response to treatment, suggesting their involvement in active HLH (74). More recently, in addition to CD8^+^/CD38^high^/HLA-DR^+^ T cell subsets, CD8^+^ T cells expressing the CD4 antigen (CD4dimCD8^+^ T cells) were found to be increased in secondary HLH and MAS. CD4dimCD8^+^ T cells were highly represented together with laboratory parameters associated with severe MAS, confirming their involvement in the pathogenesis of secondary HLH and MAS (75).

### 5.5 Cytokine level assessment, soluble CD25, and CD163 levels measurement

HLH symptoms derive from a hyperinflammatory state caused by the impaired degranulation of T cells, which fail to eliminate infected cells. The persistence of these stimuli leads to the uncontrolled production of pro-inflammatory mediators such as IFNγ, TNF-α, IL-1β, IL-6, IL-8, IL-12, IL-18, macrophage–colony-stimulating factor and hematopoietic growth factors (76). Specifically, IL-10 is produced during the activation of Th1 and Th2 cells, and of macrophages, being considered as a marker of disease activity, while IFNγ is related to HLH progression and to its complications (76). Thus, the quantification of cytokine levels is a valuable tool to monitor patients’ inflammatory status.

Among them, IL-1β, IL-6, IFNγ and IFNγ-induced chemokines, such as CXCL9, CXCL10, and CXCL11, were investigated for monitoring patients with MAS complicating sJIA through Luminex multiplexing technology (multiplexing ELISA assay). The study demonstrated increased levels of these cytokines during active MAS and secondary HLH (77).

In the last years, IL-18 and CXCL9 are being increasingly adopted in clinical settings. CXCL9 is used to monitor IFN-γ activity in HLH and MAS, in the context of IFN-γ neutralizing antibodies (i.e. emapalumab) clinical trial. Together with sCD25, it has been suggested in HLH recognition (78). Furthermore, IL-18 alone was reported to be a robust biomarker of disease activity in patients with history of MAS related to active sJIA (79, 80). IL-18 was also reported to have a role in severe/recurrent MAS with infantile enterocolitis associated with gain-of-function mutations in the NLRC4 inflammasome (NLRC4-MAS) (79). Additionally, a higher ratio of IL-18 to CXCL9 was observed in patients with MAS if compared to patients with familial or infection-associated HLH (>24 000 pg/mL, 83% sensitivity and 94% specificity) (79). Together, these results support the prognostic value of IL-18 in MAS.

Recently, a novel cytometric bead array (CBA) was developed to measure IFNγ, TNF-α, IL-2, IL-4, IL-6, and IL-10 (81). Taking advantage of this method, Tang et al. observed that HLH patients showed an increase of all cytokines but IL-2 and IL-4 during active disease if compared to healthy controls and patients with sepsis, with a restoring of basal levels during remission (76).

The increment of sCD25/sIL2r serum levels is related to immune activation in HLH (72). Ammann et al. detected elevated levels of sCD25 in FHL patients, if compared to secondary HLH samples, but they observed poor correlation with another marker of T cell activation, HLA-DR expression on CD4^+^ and CD8^+^cells (72). An interesting Japanese study proposed to consider both sCD25 levels and the sCD25/ferritin ratio to predict the presence of malignant lymphoma, since these investigations are likely to reveal an exaggerated activation of T cells (82).

The sCD25/ferritin ratio, raised the positive predictive value of 95.6%, suggesting serum sCD25 levels and sCD25/ferritin ratio as useful markers to predict the occurrence of malignant lymphoma in patients with HLH (83). However, ferritin alone is the main and widely available marker for HLH and it is essential to all the countries or settings in which specialized tests (e.g. sCD25, CXCL9) are not available.

A marker of macrophage activation is represented by soluble CD163 (sCD163) (84). High sCD163 levels indicate macrophages with marked phagocytic activity (85). In 2007, Bleesing et al. analyzed the levels of sCD25 and sCD163 in patients with sJIA complicated by MAS. By comparing these patients with a control group having MAS without sJIA, they observed that the acute phase is characterized by high levels of both molecules, comparable with those reported in FHL and secondary HLH. Due to the poor number of cases, authors suggested to test their hypothesis on a larger population to identify the cutoff levels necessary to distinguish patients at risk of developing MAS (86).

## 6 Genetic testing: from Sanger to Whole Genome Sequencing

Despite the correlation between phenotype and gene variants has been reported in HLH (44, 87), the clinical distinction among different HLH subtypes is not easy. For this reason, genetic testing remains the gold standard for the diagnosis of inherited forms (88). From 1999, the year of the first identification of an HLH-related gene with Sanger sequencing, great progress has been made.

FHL is confirmed by the presence of biallelic pathogenic variants in the genes *PRF1*, *UNC13D*, *STX11*, *STXBP2, RAB27A* and *LYST* leading to impaired cytotoxicity. Other genes involved in control of infection and dysregulated inflammasome activity have been associated to HLH as a predominant manifestation. Among these, X-linked lymphoprolipherative diseases type 1 and 2 due to hemizygous pathogenic variants in *SH2D1A* and *BIRC4* respectively, are the most known syndromes (**Table 1**). In addition to them, several inherited errors of immunity or metabolic disorders may rarely cause the HLH phenotype. Thus, extending genetic investigation over the classical FHL-related genes is recommended in the suspicion of inherited conditions (29, 88). Heterozygous pathogenic variants in FHL-related genes may be associated with severe infection and MAS in rheumatologic conditions (89, 90). Namely, heterozygous mutation predisposing to sJIA/MAS were found in *PRF1* and *UNC13D* (91–95). Even if the finding of heterozygous mutations in these patients rarely impacts the clinical management, it is worth proceeding with sequencing in these patients as well at least for research purposes.

Nowadays, several techniques are available for genetic analyses. Sanger sequencing is a targeted sequencing technique that uses dideoxyoligonucleotide to analyze specific DNA regions, and it can be considered a “first generation” sequencing method. This testing strategy counts several decades of intense application, since it is a robust technique to detect point mutations, small deletions, or duplications. High gene specificity is detrimental to the number of genes eligible for the analysis, which may be costly if compared to multiplexing testing systems. The method is fast, since it requires from a few days to a week, and less time-consuming, especially in the context of familial forms where the DNA region to be investigated is already known. Although in the last years NGS has been taking the lead, Sanger sequencing still serves as a complementary method to confirm the doubtful variants emerged and may be used to fill the gap of regions poorly covered by NGS due to GC-rich sequences or technical issues (96).

Despite the aforementioned limitations, NGS technology is a powerful method to investigate a targeted gene panel, optimized on the basis of the laboratory need in a cost-efficient manner. It may require from 2 to 6 weeks, but it can include up to 20 patients per experiment. One of the numerous examples of these platforms is the HLH sequencing panel of the Cincinnati Children’s Hospital Medical Center (CCHMC), which combines 15 HLH-associated genes and was launched in September 2013. Gadoury-Levesque et al. used this approach to perform a definite genetic diagnosis in 1892 patients with suspected HLH. The group obtained a diagnostic rate of 10.4%. This low value can be explained, by one hand, by lax inclusion criteria of patients, and on the other by the exclusion of newly reported HLH-associated genes, such as *NLRC4* and *CDC42 (* 97). Tesi et al. established a 12 HLH-associated comprehensive high-throughput platform to explain cases in which the demonstration of reduced NK cell function was not followed by a genetic diagnosis. The group reached an overall diagnostic rate of 38%, which was increased to 44% in the pediatric cohort. The mutation detection sensitivity was 97.3%, the average coverage per gene 98.0%, and the coverage of sites previously reported as mutated was over 98.6% (98).

To sum up, the NGS-based approach has high accuracy in detecting point mutations, small deletions, and insertions, which are the most frequent alterations in HLH. Notwithstanding, the method is not reliable to identify large deletions, duplications, or insertions. Considering that sequencing costs and timing are progressively decreasing, the detection of genetic causes of HLH may be more accurately provided by Whole Exome Sequencing (WES), which gives the opportunity to perform more comprehensive genetic analyses and to identify novel genes predisposing to HLH or HLH-mimicking phenotypes. WES is a method designed to perform a target resequencing of all protein-coding nuclear genes in the genome. Since non-coding sequences are generally characterized by variants with a mild or no effect on phenotype, the advantage of WES is to be directed on the small percentage of coding genome (1%) that is more likely to carry pathogenic variants (99). On the other hand, one of WES limitations is that it cannot identify structural and gross copy number variations and intronic variants affecting promoter or enhancer elements, which were reported in *UNC13D, BIRC4*, and *RAB27A* (36, 40, 100–102). This emphasizes the need for implementation of WES with specific probes, widely adopted in laboratory routine or other methods such as Whole Genome Sequencing (WGS) and RNA sequencing, which however are poorly available nowadays. Chinn et al. reported a promising use of WES in patients with HLH, in which the potential causing genes associated with HLH were identified in 58% of cases thanks to a precise patient selection by HLH-2004 criteria evaluation and a flow cytometry-based immunological screening (36).

Overall, genetic studies remain crucial for HLH diagnosis and allow familial counseling and prenatal diagnosis. The combination of immunological screenings with novel technologies, such as WES and WGS, will allow us to understand the significance of genetic variants, filling the gap between pathophysiological mechanisms and targeted treatments of HLH.

## 7 Discussion

HLH is a rare syndrome with multiple clinical manifestations. Without prompt recognition, it is invariably fatal. Despite recent improvements in survival, around 40% of patients with HLH still die from refractory disease or toxicity. Thus, a rapid and effective diagnostic approach is crucial for early treatment start and HSCT decision.

In recent years, many novel approaches have been proposed to investigate patients with clinical and laboratory findings consistent with HLH. Their rationale and reliability has been widely analyzed in this review, and some of them might gain a pivotal role in HLH management in the future. However, there are extensively validated investigations that remain now essential in the diagnostic approach. In the view of the clinical and genetic heterogeneity of the syndrome, it may still be challenging for the clinician to make quick handling decisions when facing a patient with suspected HLH. For this reason, we recommend a comprehensive diagnostic approach to HLH that integrates clinical, functional, and genetic criteria, possibly in the setting, or with the advice, of a highly specialized team (**Figure 2**). When HLH is suspected, diagnostic evaluations should immediately start to confirm the diagnosis. Since the available diagnostic criteria are non-specific for HLH, therapeutic decisions should be carefully discussed with expert physicians and individualized on each patient need. Disease severity, age at onset, underlying diseases and comorbidities need to be considered to avoid overtreatment or undertreatment. Patients with fever, splenomegaly and cytopenia should be investigated for HLH but atypical clinical presentations including newborn with acute liver failure and isolated central nervous system involvement should also be kept in mind for FHL. Hyperferritinemia is a hallmark of uncontrolled inflammation and supports the suspicion of HLH, though it is non-specific. Instead, normal ferritin levels should guide towards alternative diagnoses. Also, sCD25 and CXCL9 are increasingly used as parameters for a first HLH recognition but are still scarcely available. Patients with renal or liver failure, as well as hypofibrinogenemia can rapidly progress toward multi-organ failure indicating poor prognosis. Although not included in the HLH-2004 criteria, hyperbilirubinemia has been recently confirmed as predictor of early death (103). Among first level evaluations, bone marrow aspirate to rule out leukemia is imperative. It is also recommended to search for Leishmania, while looking for hemophagocytosis is not paramount for HLH diagnosis. First level imaging should also be performed, primarily to exclude neoplasia. In the meanwhile, etiological investigations should be initiated starting from screening for infections and rheumatological disease. Once HLH is confirmed, blood samples should be centralized to reference laboratories for functional and genetic evaluation. Indeed, flow cytometric screening of perforin expression and CD107a externalization allows a rapid (24-48h) identification of patients with FHL and guide genetic analysis (43, 51). A defective perforin expression prompt immediate Sanger investigation on *PRF1* gene, whereas a defective degranulation assay is suggestive of mutations in *UNC13D, STXBP2, STX11*, and *RAB27A*. Other diagnostic tests, including cytokines dosage, provide supporting information (i.e. high IL-18 levels for inflammasome related diseases), but are not always timely accessible for clinical decisions. Under these conditions, cytofluorimetric tests have a high diagnostic value. Cardiac evaluation with echocardiography should be performed as soon as possible both to evaluate the cardiac function and to rule out coronary signs of Kawasaki disease. Additional investigations should address rarer conditions associated to HLH including other immunodeficiencies and LPI (Lysinuric Protein Intolerance), that is the only metabolic disease really mimicking FHL.

Nevertheless, a strong clinical suspicion of genetic HLH, even without functional defects nor mutations of FHL-related genes, should direct toward NGS investigation to identify rarer subtypes of genetic HLH beyond cytotoxicity defects, such as those involving impaired control of infections or dysregulated inflammasome activation (**Table 1**). Indeed, it has been reported that only 8% of patients with suspected HLH do not carry mutations on FHL-related genes (34). Thus, in selected cases, we favor the use of WES instead of targeted resequencing panels to collect more information and, at the same time, direct the analysis towards a specific set of genes selected on a clinical basis. Furthermore, WES can also allow the identification of genetic predisposition in different conditions, including rheumatological and autoinflammatory diseases.

Thus, besides optimizing costs and timing, WES is also useful for expanding the knowledge of genetic variants associated with HLH. It is worth noting that the massive amount of information deriving from WES output should be interpreted with caution. The variants of uncertain clinical significance should be considered on a case-by-case basis, depending on the clinical phenotype, and should be validated by functional studies confirming the defect *in vitro* to clarify their biological impact. Despite WES is being increasingly adopted and easily accessible worldwide, we strongly recommend centralization of samples in reference laboratories to allow a reasoned approach starting from clinical evaluation towards functional and genetic analysis. Personnel expertise is indeed crucial to guide the diagnostic algorithm and to correctly interpret the results, with the final aim to support clinical and therapeutic decisions. Indeed, genetic findings do not always impact on patient management, including HSCT. In the last decades, great progress has been made, but novel technologies and therapeutic strategies are developing and will hopefully improve patient’s outcome in the next years.

In conclusion, as the Italian reference center for HLH, we suggest the integration between clinical evaluation, functional and genetic analysis to optimize diagnosis timing and accuracy, by following a reasoned clinical-based approach open towards novel diagnostic tools. To effectively realize this integration, we must keep in mind the knowledge collected so far by looking beyond at the same time, with the final aim to unravel the tangle of HLH spectrum, by allowing an increasingly personalized approach to each patient.

## Author contributions

ES designed the study. AC and ES wrote the manuscript. AC, LB, FP, IT, MC, and ES reviewed and edited the manuscript. All authors contributed to the article and approved the submitted version.
