# Erratum: Approaching hemophagocytic lymphohistiocytosis

**DOI:** 10.3389/fimmu.2024.1369687

**Published:** 2024-01-22

**Authors:** Frontiers Production Office

**Affiliations:** Frontiers Media SA, Lausanne, Switzerland

**Keywords:** HLH - hemophagocytic lymphohistiocytosis, FHL – familial hemophagocytic lymphohistiocytosis, perforin, degranulation, flow cytometric assay, NGS - next generation sequencing

Due to a production error, there was a mistake in affiliation 2. Instead of “Department of Pediatric Hematology Oncology, Meyer Children’s Hospital Istituto di Ricovero e Cura a Carattere Scientifico (IRCCS), Florence, Italy”, it should be “Department of Pediatric Hematology Oncology, Meyer Children’s Hospital IRCCS, Florence, Italy”.

The publisher apologizes for this mistake.

The original version of this article has been updated.

